# Integrative Hormone Metabolomic, Transcriptomic, and miRNA Sequencing Analyses Reveal Brassinosteroid-Mediated Sex Differentiation in Walnut Flower Buds

**DOI:** 10.3390/plants15152296

**Published:** 2026-07-27

**Authors:** Xia Luo, Haochang Zhao, Jiyan Tang, Zhihua Guo, Xiaoyan Lu, Jianxin Niu, Shaowen Quan

**Affiliations:** 1 College of Agriculture, Shihezi University, Shihezi 832003, China; 2 Key Laboratory of Special Fruits & Vegetables Cultivation Physiology and Germplasm Resources Utilization, Shihezi University, Xinjiang Production and Construction Group, Shihezi 832003, China

**Keywords:** walnut, sex differentiation, brassinosteroid, flower bud, hormone, MADS-MIKC, miRNA-mRNA

## Abstract

Walnut (*Juglans regia* L.) is a monoecious species with far more male than female flowers, yet the molecular mechanisms regulating sex differentiation remain largely unexplored. In this study, we investigated the effects of exogenous brassinolide (BR) on the female-to-male flower bud ratio and its regulatory mechanisms. We identified 23 April to 13 May as the critical physiological differentiation period for the ‘Xin xin 2’ variety. Exogenous application of 0.05 mg/L BR during this period significantly increased the female-to-male flower bud ratio across three consecutive years (2023–2025). Hormone metabolomics revealed that BR treatment induced sex-specific changes in ethylene and auxin contents. Transcriptomic analysis showed that BR differentially regulated MADS-MIKC genes in female and male buds. Integrated miRNA-mRNA analysis identified sex-specific regulatory modules targeting MADS-MIKC genes, including four in female buds and two in male buds. RT-qPCR and dual-luciferase assays confirmed that *jre-miR156g* directly targets the 3’-untranslated region of *JrSPL5*, with reciprocal expression patterns. Collectively, our findings demonstrate that exogenous BR increases the female-to-male flower bud ratio in walnut through multi-layered regulation involving hormone signaling, sex-specific expression of MADS-MIKC genes, and miRNA-mRNA regulatory cascades. This study provides a theoretical basis for breeding high-yielding walnut varieties by manipulating sex expression.

## 1. Introduction

Walnut is a deciduous tree bearing unisexual flowers; the pistillate (female) flowers are typically borne in terminal clusters of 2–5 flowers, whereas the staminate (male) flowers are arranged in pendulous catkins approximately 5–10 cm in length [1]. The process from flower bud differentiation to flowering in walnut takes approximately one year, spanning from March of one year to April of the next, and comprises two stages: physiological differentiation and morphological differentiation [2]. Specifically, physiological differentiation, the initial phase of flower bud differentiation, occurs from late April to early May each year [3]. Following its completion, the flower buds enter the morphological differentiation stage, which lasts from early May of the first year to April of the following year [4,5]. The flower bud differentiation cycle in walnut is relatively long, and the sex of flower buds can only be determined after they have developed to a specific external morphological stage. Recent multi-omics analyses have revealed the involvement of auxin/jasmonic acid signaling [6], stamen morphogenesis-related proteins [7], and miRNA regulation [8,9] in walnut sex differentiation. Nevertheless, the underlying molecular mechanisms remain largely unexplored.

Plant sex determination is governed by the coordinated interplay of genetic factors, phytohormones, and environmental cues [10], with participating genes broadly classified into sex-determination genes that direct reproductive organ development and sex-differentiation genes that exhibit spatiotemporally differential expression [11]. However, functionally characterized sex-determination genes remain limited and their regulatory mechanisms are highly species-specific. Classical genetic studies in model and crop species have established key paradigms for sex determination. In maize (*Zea mays*), the *Ba* and *Ts* loci govern inflorescence development [12], with the *ts4* locus encoding *miR172* that targets *APETALA2* transcription factors—a landmark example of miRNA-mediated sex regulation [13,14]. In cucumber (*Cucumis sativus*), three interacting loci—*A*/*a*, *F*/*f* and *M*/*m*—jointly determine sex phenotype by suppressing or promoting female flower development [15]. In persimmon (*Diospyros kaki*), a small RNA encoded by the *OGI* locus on the Y chromosome epigenetically targets the *MeGI* gene to coordinate female and male flower development [16,17]. Together, these cases reveal evolutionary convergence of small-RNA pathways in sex regulation across distantly related species. In *Arabidopsis*, the MADS-MIKC genes are classified into the ABCDE model, in which *AP1/SQUA* (A-class), *AP3/PI* (B-class), *AGAMOUS* (C-class), *STK/SHP* (D-class) and *SEPALLATA* (E-class) genes coordinately regulate the development of sepals, petals, stamens, carpels, and ovules [18]. Despite these findings, woody perennials remain poorly studied for sex differentiation. Walnut shows strong sexual dimorphism. Male flowers greatly outnumber female flowers. The regulatory pathways behind this imbalance are mostly unknown. Researchers have identified 52 MADS-MIKC genes in walnut [19]. Collectively, these findings highlight the diversity of sex-determination mechanisms across plant species, yet the genetic basis of sex differentiation in walnut remains largely unexplored.

Plant hormones play critical regulatory roles in sex differentiation, with distinct functions exhibited by different hormones [10]. Gibberellin primarily promotes stamen development; exogenous application increases the proportion of male flowers in spinach (*Spinacia oleracea* L.) [20] and hemp (*Rumex acetosa* L.) [21], whereas its inhibitor induces pistil development in papaya (*Carica papaya* L.) [22]. In contrast, auxin, cytokinin and ethylene mainly promote female organ development; auxin is involved in floral primordium formation and the production of female flowers in cucumber [15], cytokinin can partially restore pistil development in male kiwifruit (*genus Actinidia*) flowers [23,24], and the mechanism of ethylene in sex determination in melon (*Cucumis melo* L.) is relatively well understood, with genes such as *CmWIP1*, *CmACS7* and *CmACS11* participating in its biosynthesis and signal transduction [25,26]. Jasmonic acid and BR mainly affect male flower development, with mutants of their respective pathways exhibiting male sterility or feminisation of male flowers [27,28]. Notably, plant hormones do not function in isolation but rather form complex regulatory networks through crosstalk. For instance, BRs interact with auxin, ethylene, and salicylic acid to coordinately regulate various developmental processes [29]. Whether such hormonal crosstalk operates during sex differentiation in walnut remains unknown, although several hormones have been implicated in this process. In walnut, hormones also participate in flower bud differentiation. For instance, auxin levels increase significantly during the morphological differentiation stage [4], while gibberellin application promotes total flower production, including both female and male flowers [30].

BRs are a class of steroid hormones recognized as the sixth major class of phytohormones, widely involved in plant growth and development [31]. Since their discovery in rapeseed pollen [32], BRs have been closely associated with reproductive development, with their regulatory scope covering nearly all aspects from ovule and pollen formation to seed and fruit development [33,34]. This role is evolutionarily conserved even beyond angiosperms: a recent study in the fern *Ceratopteris richardii* demonstrated that an ortholog of the BR receptor kinase BRI1 (HER7) is required for sex determination, with BR biosynthesis up-regulated in hermaphrodites compared to male gametophytes [35]. In female reproduction, BRs promote megaspore mother cell specification by suppressing surrounding somatic cells to ensure single megaspore mother cell formation [36], and cooperate with the MADS-MIKC transcription factor *AGAMOUS* via tissue-specific regulation of the 11th C_2_H_2_ zinc finger protein to promote pistil and ovule development [37]. In male reproduction, BRs regulate pollen development and tube formation. Pollen from the BR-insensitive mutant *bri1* fails to germinate, and BR biosynthesis-deficient mutants exhibit pollen tube defects [38,39]. Key transcription factors involved in anther and pollen development, such as *SPL/NZZ*, *AMS*, *TDF1*, *MS1*, and *MS2*, are regulated by BR signaling, with recent studies uncovering additional regulatory mechanisms, including the *PP2A-BZR1-CEP1* module in pollen wall formation [40,41]. Furthermore, the *miR156-SPL* module is a highly conserved and critical regulator of reproductive development in plants. In *Arabidopsis*, *AtSPL8* affects anther development [42], while *AtSPL3*, *AtSPL4*, and *AtSPL5* promote the transition of floral meristem identity [43]. In walnut, *miR156* targets *JrSPL02* to promote early flowering [44], and in yellowhorn (*Xanthoceras sorbifolium* Bunge.), the miR156-SPL module promotes female flower differentiation [45]. Given the central role of SPL transcription factors in both BR signaling and reproductive development, we hypothesized that the miR156-SPL module may mediate BR-regulated sex differentiation in walnut.

China has the largest walnut cultivation area in the world, with approximately 371,689 hectares and in-shell walnut production of 1.5 million metric tons in 2024 (FAOSTAT). Therefore, achieving high walnut yield has long been a primary goal for researchers and growers. Moreover, the yield of walnut is closely related to the number of flower buds [46]; excessive pollen can even lead to flower abortion [47,48]. Among various plant growth regulators, BRs have attracted considerable attention due to their critical role in plant sex differentiation. BRs can both promote pistil and ovule development [34,49] and regulate pollen fertility and pollen tube growth [39,40], and their exogenous application significantly affects sex expression in multiple crops [50]. Therefore, the aim of this study was to investigate the molecular mechanisms by which exogenous BR application during the critical period of flower bud differentiation increases the female-to-male flower bud ratio in walnut, thereby providing a foundation for breeding high-yielding varieties.

## 2. Results

### 2.1. Physiological Differentiation of Walnut Flower Buds

To determine the timing of the physiological differentiation phase in walnuts, observations were conducted on walnut flower buds, with the first observation day recorded as Day 1. On Day 1, the current-year shoots of the walnut tree were still elongating (Figure 1A), and no characteristics of sexual differentiation were observed in the paraffin sections. The axillary buds of the walnut appeared dome-shaped (Figure 1B). On Day 7, the axillary buds on the shoots could not be distinguished by sex with the naked eye (Figure 1C), but protrusions of male flower bud growth points were observed in the paraffin sections (Figure 1E). On Day 14, the sex of the female and male flower buds on the current-year shoots could be distinguished: the female flower buds were dome-shaped, while the male flower buds exhibited a pagoda-like shape. At this stage, the growth point of the female flower buds appeared as a flat surface with tightly arranged cells (Figure 1G), while the inner stamen primordia of the male flower buds gradually became prominent (Figure 1H). On Day 27, the characteristics of the female and male flower buds on the shoots became more distinct: the growth points of the female flower buds began to protrude upward (Figure 1J), and the stamen primordia differentiated into anthers. Walnut axillary buds require approximately 27 days to differentiate to the stage at which female and male flower buds become distinguishable by the naked eye, and this period constitutes the physiological differentiation phase of walnut flower buds.

### 2.2. The Effect of BRs on the Female/Male Flower Bud Ratio

To investigate the effects of different concentrations of BR on the differentiation of female and male flower buds in walnut, different concentrations of BR were sprayed onto the trees when the new shoots began to grow in 2023. The influence of different BR concentrations on the female-to-male flower bud ratio of ‘Xin xin 2’ showed an initial increase followed by a decrease. Among the treatments, compared with the control, 0.05 mg/L BR significantly increased the female-to-male flower bud ratio of ‘Xin xin 2’, with the ratio rising by 73.8% (Figure 2A). In 2024 and 2025, 0.05 mg/L BR was applied when the current-year shoots started to grow. Compared with the control, BR application significantly enhanced the female-to-male flower bud ratio of ‘Xin xin 2’ in both years: by 44.7% in 2024 and by 122.3% in 2025 (Figure 2B).

### 2.3. Alteration of Plant Hormone Content by BR Spraying in Walnut Leaves and Flower Buds

To investigate the effects of BR on endogenous hormone levels, we measured the contents of seven phytohormones in walnut leaves, female flower buds, and male flower buds. These included jasmonic acid, ethylene, abscisic acid, gibberellin A3, auxin, salicylic acid, and BR. Metabolomic analysis was conducted on BR-treated and control groups from different tissue sources (male flower buds, female flower buds, and leaves). As shown in Figure 3, in leaves, ethylene increased significantly after BR application, while gibberellin A3 and salicylic acid decreased significantly. In female flower buds, ethylene, auxin and salicylic acid increased significantly. In male flower buds, auxin, and salicylic acid increased significantly, while ethylene content decreased significantly. The contents of abscisic acid, gibberellin A3, and BR in female and male flower buds showed no significant changes. This indicates that multiple endogenous hormones—including ethylene, auxin, and salicylic acid—may be involved in the response to exogenous BR application.

### 2.4. Identification of Changes in the Transcriptomic Profile of Walnut Flower Buds

To investigate the effects of BR spraying on gene expression in walnut, transcriptome sequencing was performed on leaves, female flower buds, and male flower buds from both control and BR-treated plants. In the comparison between BR-treated female flower buds and control female flower buds, a total of 4899 differentially expressed genes were identified, among which 1896 were up-regulated and 3003 were down-regulated. In the comparison between BR-treated male flower buds and control male flower buds, a total of 3816 differentially expressed genes were screened, with 1851 up-regulated and 1965 down-regulated (Figure 4A). Of these, 2634, 2621 and 1706 differentially expressed genes exhibited tissue-specific expression in leaves, female flower buds and male flower buds, respectively (Figure 4B). KEGG enrichment analysis revealed the significantly enriched pathways of differentially expressed genes in female and male flower buds. As shown in Figure 4C, the top five most significantly enriched pathways in female flower buds were plant–pathogen interaction, plant hormone signal transduction, the MAPK signaling pathway in plants, ABC transporters, and flavonoid metabolism. As shown in Figure 4D, the top five most significantly enriched pathways of differentially expressed genes in male flower buds were photosynthesis, starch and sucrose metabolism, the MAPK signaling pathway in plants, plant hormone signal transduction, and flavonoid metabolism.

### 2.5. Identification of Transcription Factors from Differentially Expressed Genes and Expression Patterns of the MADS-MIKC Gene Family in Female and Male Flower Buds After BR Treatment

As shown in Figure 5A, 19 transcription factor families were differentially expressed in female flower buds after BR treatment. Among them, 13 families showed a fold enrichment greater than 1. The MYB family exhibited the highest fold enrichment, followed by the MADS-MIKC family, with the ARF and Dof families tied for third place. In male flower buds, 19 transcription factor families were differentially expressed in response to BR treatment. Among them, 14 families displayed a fold enrichment greater than 1. The MADS-MIKC family showed the highest fold enrichment, followed by the MYB and WRKY families. Notably, the MADS-MIKC gene family showed high fold enrichment changes in both female and male flower buds. This suggests that this key floral organ identity regulator family may play a significant role in sex-biased differentiation patterns in walnut.

As shown in Figure 5B, the expression patterns of differentially expressed MADS-MIKC genes in female and male flower buds are presented. In female flower buds, ten differentially expressed genes were identified, including four AP1/SQUA (*JrMADS5*, *JrMADS8*, *JrMADS14*, *JrMADS23*), two SOC1 (*JrMADS16*, *JrMADS18*), and one each from AGL17 (*JrMADS6*), AP3/PI (*JrMADS19*), and SEP (*JrMADS39*). Among these, *JrMADS6* and *JrMADS16* were down-regulated, while the other eight were up-regulated. In male flower buds, thirteen differentially expressed genes were detected, including four AP1/SQUA (*JrMADS5*, *JrMADS8*, *JrMADS14*, *JrMADS40*), three SVP (*JrMADS1*, *JrMADS37*, *JrMADS41*), two SEP (*JrMADS28*, *JrMADS39*), two SOC1 (*JrMADS16*, *JrMADS18*), and one each from AGL17 (*JrMADS52*) and AGL6 (*JrMADS48*). Four genes were down-regulated (*JrMADS37*, *JrMADS41*, *JrMADS52*, and *JrMADS16*), and the remaining nine were up-regulated.

### 2.6. Identification of Changes in the miRNA-Seq of Walnut Flower Buds

To investigate the effects of exogenous BR on miRNA expression in walnut floral buds, miRNA sequencing was performed on leaves, female flower buds, and male flower buds from both control and BR-treated plants. Compared with their respective controls, 109 differentially expressed miRNAs (targeting 2712 genes) were identified in BR-treated female flower buds and 138 (targeting 1990 genes) in BR-treated male flower buds (Figure 6A). Tissue-specific analysis showed 91, 72, and 68 differentially expressed miRNAs in leaves, female flower buds, and male flower buds, respectively (Figure 6B). KEGG enrichment analysis of the target genes of these miRNAs revealed that “Starch and sucrose metabolism” was the most enriched pathway in both female and male flower buds (Figure 6C,D).

### 2.7. Identification of miRNA-mRNA Regulatory Modules Targeting the MADS-MIKC Gene Family

Through integrated transcriptomic and miRNA-seq analysis, we identified miRNA-mRNA regulatory modules targeting the MADS-MIKC gene family in female and male flower buds. As shown in Figure 7A, four differentially expressed miRNA-mRNA modules were identified in female flower buds: *jre-miR156g-JrSPL2/5/6/24*, *jre-miR160b-JrARF11/24*, *jre-miR167b-5p-JrARF8*, and *jre-miR319a-JrTCP4-like*. These four modules targeted eight MADS-MIKC genes, including four AP1/SQUA (*JrMADS5/8/14/23*), two SOC1 (*JrMADS16/18*), one AP3/PI (*JrMADS19*), and one SEP (*JrMADS39*). As shown in Figure 7B, two differentially expressed modules were identified in male flower buds: *jre-miR156g-JrSPL18/24* and *jre-miR160b-JrARF11*. These two modules targeted thirteen MADS-MIKC genes, including four AP1/SQUA (*JrMADS5/8/14/40*), three SVP (*JrMADS1/37/41*), two SEP (*JrMADS28/39*), two SOC1 (*JrMADS16/18*), one AGL17 (*JrMADS52*), and one AGL6 (*JrMADS48*). In female flower buds, *JrTCP4-like* (9.7-fold), *JrARF11* (6.5-fold), *JrSPL2* (5.1-fold), *JrARF8* (4.5-fold), *JrSPL5* (3.5-fold), and *JrSPL6* (3.4-fold) were all significantly up-regulated after BR treatment (>3-fold). In male flower buds, only *JrSPL18* (1.3-fold) and *JrSPL24* (2.0-fold) showed moderate up-regulation. These results indicate that BR induces a stronger transcriptional response in female flower buds than in male flower buds within these core regulatory modules.

### 2.8. jre-miR156g Targets and Cleaves the 3’-Untranslated Region of JrSPL5

To further validate the regulatory relationship between *jre-miR156g* and *JrSPL5*, we performed RT-qPCR analysis to examine their expression patterns in leaves, female flower buds, and male flower buds across different developmental stages. As shown in Figure 8A, *jre-miR156g* exhibited its highest expression level in leaves at Day 1, followed by a gradual decline over time, with only relatively low expression detected in both female and male flower buds. In contrast, *JrSPL5* displayed a reciprocal expression pattern, showing the highest transcript accumulation in female and male flower buds, and its expression was significantly up-regulated following BR treatment (Figure 8B). To precisely determine the cleavage site, we conducted local BLAST alignment between the mature *jre-miR156g* sequence and the *JrSPL5* transcript using NCBI BLAST+ (version 2.12.0+) on an Ubuntu Linux system, which indicated that the target site was located within the 3’-untranslated region of *JrSPL5* (Figure 8C). We further cloned two precursor variants of *jre-miR156g* (*jre-miR156g.2* and *jre-miR156g.4*) and performed dual-luciferase assays by co-transforming these precursors with the 3’-untranslated region of *JrSPL5* into tobacco leaves. The results showed that both j*re-miR156g.2* and *jre-miR156g.4* effectively targeted and cleaved the 3’-untranslated region of *JrSPL5*, leading to a marked decrease in the LUC/REN ratio (Figure 8D,E). Collectively, these results confirm that *jre-miR156g* directly cleaves the 3’-untranslated region of *JrSPL5*.

## 3. Discussion

The sex of walnut flower buds is determined at the very beginning of differentiation—the critical period of physiological differentiation. Precise identification of this temporal window is therefore essential for understanding and manipulating the sex differentiation process. Based on morphological and anatomical observations,20 April–4 May was designated as the physiological differentiation stage for female flower buds of *Juglans sigillata* Dode (the ‘Qianhe 7’ variety) [3]. Consistent with these findings, in the present study, 23 April–13 May was identified as the physiological differentiation stage for the ‘Xin xin 2’ walnut variety, representing a key period for regulating the differentiation of male and female flower buds.

Exogenous application of plant growth regulators is an effective approach for modulating sex expression. For instance, spraying gibberellins on walnut trees can increase the number of male flowers [30]. In this study, we found that BR application during the physiological differentiation stage significantly increased the female-to-male flower bud ratio in walnut. BRs are plant steroid hormones. Since their discovery in rapeseed pollen, they have been closely associated with reproductive traits, including the regulation of ovule number [34], anther formation [40], pollen tube elongation [50], and megaspore mother cell specification [33]. However, the mechanisms by which BR regulates sex differentiation in walnut remain unclear. Our results demonstrate that BR triggers tissue-specific hormonal changes: ethylene and auxin increased in female flower buds, while ethylene decreased but auxin increased in male flower buds. It is well established that ethylene and auxin promote female flower development in cucurbits [15,51]. Recent studies further revealed a reciprocal relationship between these two hormones during sex determination, in which ethylene promotes carpel formation through auxin at the early stage, and auxin subsequently boosts ethylene biosynthesis in the carpel to inhibit stamen development [52]. Thus, the BR-induced accumulation of ethylene and auxin in female flower buds, together with ethylene suppression in male buds, may create a hormonal environment favoring female differentiation while restraining male development. These findings suggest that ethylene and auxin are key downstream signals mediating the effect of exogenous BR on sex differentiation in walnut.

Beyond hormonal changes, BR treatment induced widespread transcriptional reprogramming. Transcriptome analysis revealed 4899 differentially expressed genes in female flower buds and 3816 in male flower buds. Among the differentially expressed genes, MADS-box genes were prominently represented. These genes are central to floral organ identity [18,19]. In female flower buds, ten MADS-MIKC genes (eight up-regulated, two down-regulated) responded to BR treatment, including AP1/SQUA (*JrMADS5*, *JrMADS8*, *JrMADS14*, *JrMADS23*), SOC1 (*JrMADS16*, *JrMADS18*), AGL17 (*JrMADS6*), AP3/PI (*JrMADS19*), and SEP (*JrMADS39*). Notably, the up-regulation of AP3/PI (B-class) and SEP (E-class) genes is noteworthy; B-class genes specify petal and stamen identity, while E-class genes act as co-factors to determine floral organ identity [53]. E-class genes act as co-factors with other MADS-MIKC proteins to determine floral organ identity [54]. The up-regulation of SVP genes in male flower buds is intriguing. SVP genes are known floral repressors in *Arabidopsis* [55]. Their up-regulation may delay or modulate male organ development. This may contribute to the shift toward female differentiation under BR treatment. The differential expression of MADS-MIKC genes in female versus male flower buds suggests that BR regulates sex differentiation through sex-specific modulation of these key floral regulators.

Sex differentiation in plants is also associated with miRNAs [56]. For example, a Y chromosome-encoded miRNA acts as a sex-determining factor in persimmon [16], and the *miR157/SPL* axis regulates floral organ growth and ovule development by modulating MADS-MIKC genes and auxin signaling in *Gossypium hirsutum* [57]. In previous studies, miRNAs from 45 families were found to be expressed in female and male flower buds of walnut, with *miR156* involved in the transition of flowering time [8]. The *miR156-SPL* module has also been shown to regulate the juvenile-to-adult phase transition and reproductive phase in *Populus tomentosa* [58]. In this study, integrated transcriptomic and miRNA-seq analysis identified sex-specific miRNA-mRNA regulatory modules that directly target MADS-MIKC genes. In female flower buds, four modules—*jre-miR156g-JrSPL2/5/6/24*, *jre-miR160b-JrARF11/24*, *jre-miR167b-5p-JrARF8*, and *jre-miR319a-JrTCP4-like*—target eight MADS-MIKC genes, including AP1/SQUA (A-class), AP3/PI (B-class), and SOC1. Notably, the female-specific *miR319-TCP* module may repress stamen development [59]. *miR160-ARF* and *miR167-ARF* modules link BR-induced miRNA changes to auxin signaling, consistent with increased auxin content in female buds after BR treatment [60]. In male flower buds, only two modules—j*re-miR156g-JrSPL18/24* and *jre-miR160b-JrARF11*—target thirteen MADS-MIKC genes, including SVP floral repressors, SEP (E-class), and AGL6. The targeting of SVP genes, known floral repressors in *Arabidopsis* [55], is particularly intriguing. Their up-regulation may delay male organ development and contribute to the shift toward female differentiation. Thus, BR induces sex-specific miRNA-mRNA-MADS regulatory cascades: female buds activate four modules targeting B-class and E-class genes to promote female development, while male buds activate two modules targeting SVP repressors to attenuate male development. These cascades provide a mechanistic link between BR application and the increased female-to-male flower bud ratio in walnut.

Furthermore, among the core genes responsive to BR, five target genes of *jre-miR156*, *JrS*PL2/5/6/18/24, were identified, constituting the *miR156-JrSPL* regulatory module. The *miR156-SPL* module is highly conserved in plants and is widely involved in reproductive development by regulating the expression of *SPL* transcription factors [48,61,62]. For example, this module is involved in ovary and fruit development in tomato(*Solanum lycopersicum*), regulates flowering time in citrus(*Citrus* spp.), affects male fertility in *Arabidopsis*, and controls inflorescence development in maize(*Zea mays*) [63,64,65,66]. In walnut, *miR156* targets *JrSPL02* to promote early flowering [44]. In this study, we validated that *jre-miR156g* directly targets the 3’-untranslated region of *JrSPL5* via dual-luciferase assays, and its expression was negatively correlated with *JrSPL5* across tissues, with *JrSPL5* being significantly up-regulated by BR. Given these findings, we propose that the jre-*miR156g-JrSPL* module may play a key role in sex differentiation of walnut flower buds.

## 4. Materials and Methods

### 4.1. Paraffin-Embedded Sections

The collected flower bud samples were first fixed with FAA (70%) fixative. After removing the outer scales from the female flower buds, both female and male buds were softened by soaking them in glycerol. They were then sequentially dehydrated and cleared through a graded series of ethanol (70%, 85%, 95% and 100%), followed by a mixture of absolute ethanol and xylene (1:1, *v*/*v*), and then infiltrated with liquid paraffin at 60 °C for 36 h. Finally, the samples were embedded, sectioned using a LEICA CM1950 microtome (Leica Biosystems, Wetzlar, Germany), mounted on slides, baked, stained with safranin O and fast green, sealed with coverslips, and examined and photographed under a microscope.

### 4.2. Screening of Suitable BR Concentrations and Sample Collection

The walnut cultivar ‘Xin xin 2’ (25-year-old) planted in Wensu County, Xinjiang, with a spacing of 5 m × 5 m, was used as the experimental material, and the study was conducted from 2023 to 2025. All spraying was performed at 9:00 AM (local time, UTC + 8) until runoff; with a spray volume of approximately 3 L per tree; BR was first dissolved in anhydrous ethanol as a stock solution (1 mg/mL) and then diluted with distilled water to the final concentrations, with the control treated with the same volume of anhydrous ethanol diluted in distilled water, and Tween-20 (0.05%, *v*/*v*) was added as a surfactant to all spray solutions. In 2025, based on preliminary anatomical observations, the critical physiological differentiation period for this cultivar was identified as 23 April to 13 May (Figure 1), during which eight BR concentrations (0, 0.01, 0.05, 0.1, 0.5, 1, 1.5 and 2 mg/L) were sprayed to screen for the optimal concentration; samples of leaves, female flower buds, and male flower buds were collected on 13 May (Day 27) from trees treated with 0.05 mg/L BR and from control trees, and these samples were used for transcriptome sequencing, miRNA sequencing, and hormone metabolomics analysis. In 2024, the selected optimal concentration (0.05 mg/L) was sprayed on 10 April, and the numbers of female and male flower buds were recorded in June to evaluate the effect of BR on the female-to-male flower bud ratio; female and male flower buds were distinguished by external morphology (female buds dome-shaped, male buds pagoda-like), and for each tree, the number of female and male flower buds was counted from 10 randomly selected branches in each of the four cardinal directions (east, south, west, and north), with the female-to-male ratio subsequently calculated. In 2025, the optimal concentration (0.05 mg/L) was sprayed on 17, 23, and 30 April; samples of leaves, female flower buds, and male flower buds were collected on 23 April (Day 7), 30 April (Day 14), and 13 May (Day 27), with the samples collected on 23 April and 30 April used for paraffin sectioning to track developmental progression and for RT-qPCR analysis of *jre-miR156g* and *JrSPL5* expression patterns, and the samples collected on 13 May used for paraffin sectioning, RT-qPCR validation, and cloning of *jre-miR156g* precursors and the 3’-untranslated region of *JrSPL5*; all samples were immediately frozen in liquid nitrogen and stored at −80 °C until use. For all experiments, three biological replicates were performed, each consisting of six walnut trees, for a total of 18 trees per treatment group.

### 4.3. Determination of Hormone Content

Compounds were extracted from walnut tissues and separated using a Waters ACQUITY I-Class UPLC system (Waters Corporation, Milford, MA, USA) with an ACQUITY UPLC HSS T3 column (Waters Corporation, Milford, MA, USA) (100 × 2.1 mm, 1.8 μm). The mobile phase consisted of 0.1% formic acid in water (A) and 0.1% formic acid in acetonitrile (B). The column temperature was 40 °C, the autosampler was 10 °C, and the injection volume was 5 μL. Mass spectrometry was performed using a SCIEX QTRAP 6500+ triple quadrupole mass spectrometer (SCIEX, Framingham, MA, USA) with an IonDrive Turbo V ESI source in MRM mode. The contents of jasmonic acid, ethylene (1-aminocyclopropanecarboxylic acid), abscisic acid, gibberellin A3, auxin (indole-3-acetic acid), and salicylic acid were analyzed via UHPLC-MS/MS. Concentrations were quantified using external standard curves.

### 4.4. Transcriptome Sequencing and Gene Functional Analysis

Total plant RNA was extracted using the RNAprep Pure Plant Kit (Tiangen, Beijing, China). RNA concentration and purity were measured with a NanoDrop 2000 (Thermo Fisher Scientific, Waltham, MA, USA), and integrity was assessed using the RNA Nano 6000 Assay Kit on an Agilent Bioanalyzer 2100 system (Agilent Technologies, Santa Clara, CA, USA). Subsequently, 1 µg of total RNA per sample was used as input to prepare sequencing libraries with the Hieff NGS Ultima Dual-mode *mRNA* Library Prep Kit for Illumina (Yeasen Biotechnology, Shanghai, China). The procedure involved mRNA enrichment using poly-T oligo-attached magnetic beads, double-stranded cDNA synthesis, end repair and A-tailing, adapter ligation with sample indices, and PCR amplification. Library purification was performed with the AMPure XP system (Beckman Coulter, Brea, CA, USA), and quality was verified on the Agilent Bioanalyzer 2100. Qualified libraries were sequenced on an Illumina NovaSeq platform (Illumina, San Diego, CA, USA) to generate 150 bp paired-end reads.

For data analysis, gene expression levels were quantified using FPKM. Differential expression analysis between groups was conducted with DESeq2 (version 1.42.0), applying a negative binomial model and the Wald test, with significance thresholds set at |fold change| ≥ 2 and FDR < 0.01. Transcription factors among the differentially expressed genes were identified using the Plant Transcription Factor Database (PlantTFDB version 5.0, http://planttfdb.gao-lab.org/, accessed on 17 July 2026), based on the walnut genome annotation. To evaluate whether specific transcription factor families were overrepresented among the differentially expressed genes, fold enrichment was calculated as (D/C)/(B/A), where D is the number of DEGs in a given TF family, C is the total number of TFs in that family, B is the total number of DEG TFs across all families, and A is the total number of TFs in the walnut genome. A fold enrichment greater than 1 indicates overrepresentation. The total numbers of TFs in each family were obtained from PlantTFDB v5.0. Functional enrichment analysis of DEGs was performed via GO terms using the clusterProfiler package (version 4.10.0) (based on the Wallenius non-central hypergeometric distribution) and KEGG pathways using the KOBAS database and clusterProfiler. The raw transcriptome sequencing data are not publicly available at this stage due to their use in ongoing unpublished studies. All processed expression data are provided as Supplementary Materials.

### 4.5. MiRNA Sequencing and Functional Analysis

Total RNA was extracted using TRIzol reagent (Invitrogen, Carlsbad, CA, USA), and its integrity, purity and concentration were assessed via agarose gel electrophoresis and Nanodrop measurement (Thermo Fisher Scientific, Waltham, MA, USA). Sequencing libraries were constructed using the TruSeq Small RNA Library Prep Kit (Illumina, San Diego, CA, USA) involving steps such as 3′ and 5′ adapter ligation, reverse transcription, PCR amplification, and fragment selection by PAGE gel electrophoresis. After passing quality control, the libraries were subjected to single-end sequencing on the Illumina NovaSeq 6000 platform (Illumina, San Diego, CA, USA). Raw sequencing data were quality-filtered using fastp (version 0.23.4) to obtain clean reads of 18–30 nt in length. Subsequently, Bowtie (version 1.3.1) was used to align the clean reads against the Silva, GtRNAdb, Rfam and Repbase databases to remove non-coding RNAs and repetitive sequences. The remaining reads were then aligned to the reference genome and the miRBase database (release 22) via miRDeep2 (version 2.0.1.3) to identify known miRNAs and predict novel miRNAs. Following expression quantification, differential expression analysis was performed using DESeq2 (version 1.42.0), with the significance threshold set at |log_2_FC| ≥ 1 and FDR ≤ 0.01. Target genes of differentially expressed miRNAs were predicted using the miRBase database. Only genes that were both predicted as miRNA targets and identified as differentially expressed in our transcriptome data (|log_2_FC| ≥ 1, FDR ≤ 0.01) were retained as high-confidence target genes. GO functional enrichment and KEGG pathway enrichment analyses were conducted on the predicted target genes of differentially expressed miRNAs using the ClusterProfiler (version 4.10.0) and KOBAS software. To further explore the transcriptional regulation of MADS-MIKC genes by transcription factors within these modules, motif analysis was performed using FIMO (version 5.5.2) with the JASPAR core plant motif database (version 2024). The regulatory networks were visualized using Cytoscape (version 3.10.2). The raw miRNA sequencing data are not publicly available at this stage due to their use in ongoing unpublished studies. All processed miRNA expression data and target gene lists are provided as Supplementary Materials.

### 4.6. RT-qPCR

Total RNA was extracted from walnut flower buds using a Plant RNA Extraction Mini Kit (Ke Di Yuan, Xinjiang, China). Primers for *JrSPL5* were designed using the NCBI Primer Design Tool (NCBI, Bethesda, MD, USA). Reverse transcription and qPCR for *JrSPL5* were performed using the TransScript One-Step gDNA Removal and cDNA Synthesis SuperMix kit (Beijing TransGen Biotech, Beijing, China). Primers for *jre-miR156g* were designed using the Vazyme small-RNA primer design tool. Reverse transcription and qPCR for jre-miR156g were performed using Vazyme-specific kits (Nanjing Vazyme Biotech Co., Ltd., Nanjing, China). All primers were synthesized by Youkang Biology (Beijing, China). The relative expression levels were calculated using the 2^−ΔΔCt^ method. Walnut Actin was used as the internal reference gene. All reactions were performed with three biological replicates and three technical replicates.

### 4.7. Dual-Luciferase Reporter Assay for jre-miR156g Targeting JrSPL5

To validate the regulatory relationship between *jre-miR156g* and *JrSPL5*, the mature sequence of *jre-miR156g* was aligned with the *JrSPL5* transcript, and a complementary target site was identified within the 3′-untranslated region of *JrSPL5*. Two precursor variants of *jre-miR156g* (*jre-miR156g.2* and *jre-miR156g.4*) were cloned into the Pgreen II 62SK vector (Shanghai Weidi Biotechnology Co., Ltd., Shanghai, China). The 3′ UTR fragment of *JrSPL5* containing the predicted *jre-miR156* binding site was cloned into the Pgreen II 0800-miRNA vector (Coolaber, Beijing, China). All recombinant plasmids were verified by Sanger sequencing and then transformed into *Agrobacterium tumefaciens* strain GV3101 (pSoup-p19) (Weidi Biotechnology, Shanghai, China). *Nicotiana benthamiana* plants were grown in a growth chamber at 25 °C under a 16 h light/8 h dark photoperiod. The transformed agrobacteria were cultured to an OD600 of 0.6–0.8 and infiltrated into N. benthamiana leaves using a needleless syringe. After infiltration, the plants were kept in the dark for 24 h and then incubated under low light for two days. Firefly luciferase (LUC) and Renilla luciferase (REN) activities were measured using a dual-luciferase assay kit (Coolaber) with a GloMax 20/20 Luminometer (Promega Corporation, Madison, WI, USA). The LUC/REN ratio was calculated to evaluate the cleavage efficiency. Each construct was tested with three biological replicates.

### 4.8. Statistical Analysis

All data are presented as mean ± standard deviation (SD) of three biological replicates. Statistical significance was determined using one-way ANOVA followed by Fisher‘s LSD test for multiple comparisons, or two-way ANOVA for comparisons involving two factors, as indicated in the figure legends. All statistical analyses were performed using R (version 4.3.0) with the appropriate packages. Figures were generated using the ggplot2 (version 3.4.2) package in R. A *p*-value < 0.05 was considered statistically significant.

## 5. Conclusions

In this study, we first identified 23 April to 13 May as the critical physiological differentiation period for the ‘Xin xin 2’ walnut variety. Based on this finding, we demonstrated that exogenous application of 0.05 mg/L BR during this period significantly increased the female-to-male flower bud ratio. To investigate the underlying mechanisms, we examined hormone contents, transcriptomic profiles, and miRNA expression in female and male flower buds. BR treatment induced tissue-specific changes in endogenous hormones, particularly ethylene and auxin, which showed differential responses between female and male buds. Transcriptomic analysis further revealed that BR altered the expression of MADS-MIKC genes in a sex-specific manner. Moreover, integrated miRNA-mRNA analysis identified four regulatory modules in female flower buds and two in male flower buds that target MADS-MIKC genes. Among these, the *jre-miR156g-JrSPL5* module was experimentally validated, showing reciprocal expression patterns and direct targeting of the 3’-untranslated region of *JrSPL5*. Collectively, these findings provide a multi-layered regulatory framework for understanding BR-mediated sex differentiation in walnut and lay a foundation for breeding high-yielding varieties through sex ratio manipulation.

## Figures and Tables

**Figure 1:**
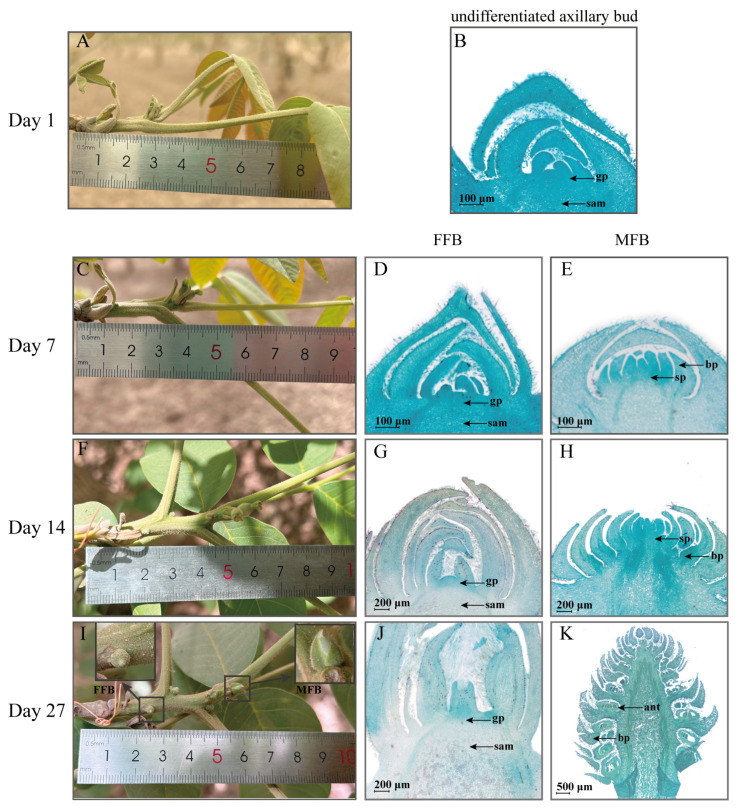
Developmental process of the critical period of walnut physiological differentiation. Observations were made on Day 1, Day 7, Day 14, and Day 27 (18 April, 23 April, 30 April, and 13 May 2025, respectively) during the physiological differentiation period of walnut. (**A**,**B**): Walnut shoot and axillary bud on Day 1. (**C**–**E**): Walnut shoot, female flower bud, and male flower bud on Day 7. (**F**–**H**): Walnut shoot, female flower bud, and male flower bud on Day 14. (**I**–**K**): Walnut shoot, female flower bud, and male flower bud on Day 27. The paraffin sections were stained with safranin O (safranin T) and fast green, with red indicating lignified tissues and green indicating cytoplasmic and cellulose wall structures. Abbreviations: FFB, female flower bud; MFB, male flower bud; gp, growth point; sam, shoot apical meristem; sp, stamen primordium; bp, bract primordium; ant, anther.

**Figure 2:**
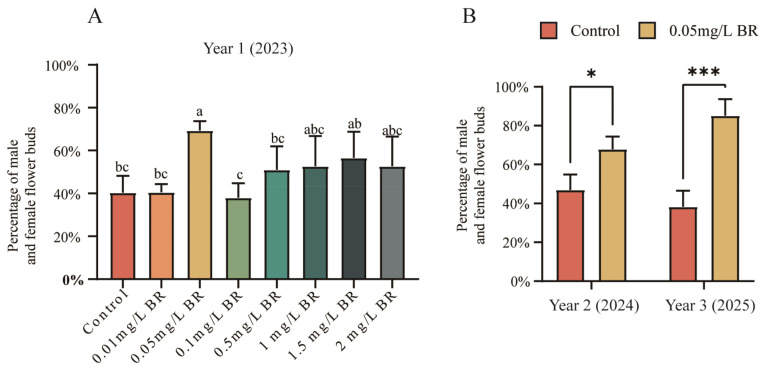
The effect of BR spraying on the female-to-male flower bud ratio. (**A**) BR (0, 0.01, 0.05, 0.1, 0.5, 1, 1.5, and 2 mg/L) was sprayed onto the walnut trees on 15 April 2023 (year 1), and the ratio of female to male flower buds was recorded on 5 June. Data are presented as mean ± SD (*n* = 3). Different letters (a, b, c) above the bars indicate significant differences at *p* < 0.05 (one-way ANOVA, Fisher’s LSD test). (**B**) On 10 April 2024 (year 2), walnut trees were sprayed with 0.05 mg/L BR; the ratio of female to male flower buds was recorded on 30 May. On 17 April, 23 April and 30 April 2025 (year 3), the trees were sprayed with 0.05 mg/L BR; the ratio of female to male flower buds was recorded on 13 May. Asterisks indicate significant differences between the control and BR treatment groups for each year (*** *p* < 0.001, * *p* < 0.05; Two-way ANOVA). Abbreviations: BR, brassinolide.

**Figure 3:**
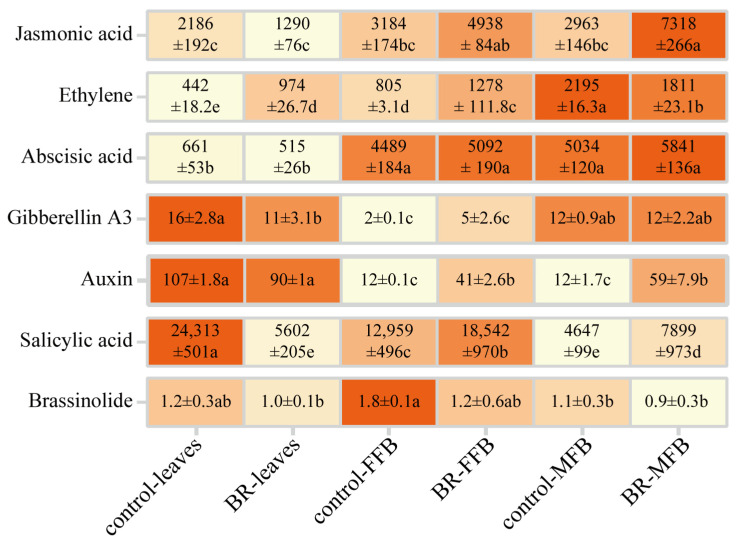
Heatmap of endogenous hormone contents in walnut leaves and flower buds after treatment with 0.05 mg/L BR, including seven phytohormones: jasmonic acid, ethylene, abscisic acid, gibberellin A3, auxin, salicylic acid, and brassinolide (units: ng/g, fresh weight). Different colors represent hormone abundance levels, with orange indicating high content and yellow indicating low content. Different letters (a, b, c, d, e) above the bars indicate significant differences at *p* < 0.05 (one-way ANOVA, Fisher’s LSD test). Abbreviations: BR, brassinolide; FFB, female flower bud; MFB, male flower bud.

**Figure 4:**
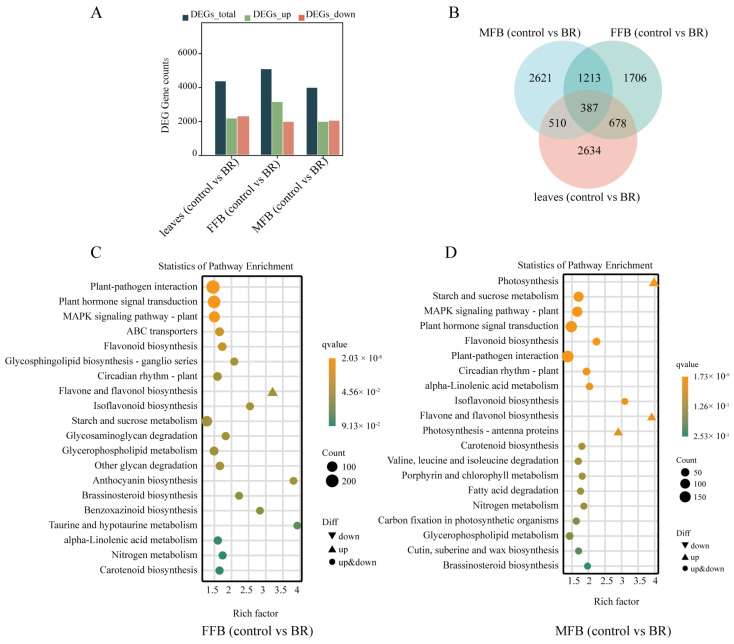
Screening of differentially responsive genes in female and male walnut flower buds after BR application. (**A**) Number of differentially expressed genes in leaves, female flower buds, and male flower buds after BR treatment (0.05 mg/L). (**B**) Venn diagram showing tissue-specific and overlapping differentially expressed genes among leaves, female flower buds, and male flower buds. (**C**) KEGG pathway enrichment analysis of differentially expressed genes in female flower buds. (**D**) KEGG pathway enrichment analysis of differentially expressed genes in male flower buds. Differentially expressed genes were identified with |log_2_(fold change)| ≥ 2 and false discovery rate (FDR) < 0.01. Only pathways with *p* < 0.01 are shown in (**C**,**D**). Abbreviations: FFB, female flower bud; MFB, male flower bud.

**Figure 5:**
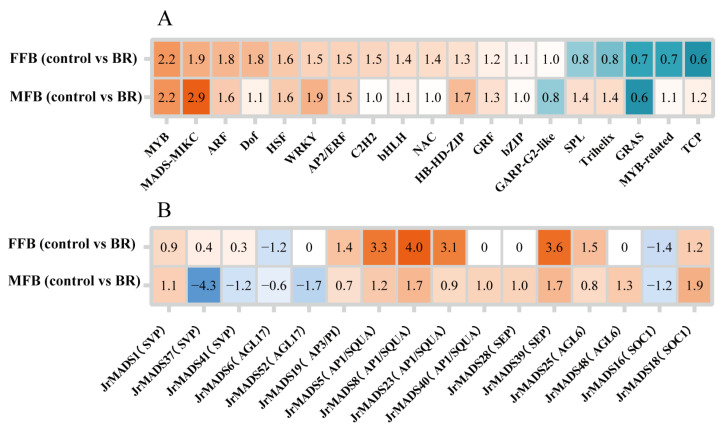
Transcription factor families enriched in female and male flower buds after BR treatment and expression patterns of the MADS-MIKC gene family. (**A**) Fold enrichment of differentially expressed transcription factors (DEG TFs) in major families in female and male flower buds after BR treatment. The number above each bar indicates the absolute count of DEG members in that family. Enrichment was calculated as (D/C)/(B/A), where D = DEG TFs in a given family, C = total TFs in that family, B = total DEG TFs across all families, and A = total TFs in the walnut genome. (**B**) Heatmap showing the expression patterns of differentially expressed MADS-MIKC genes in female and male flower buds. The color scale represents the log_2_ (fold change) values, with red indicating up-regulation and blue indicating down-regulation. Differentially expressed genes were identified with |log_2_FC| ≥ 2 and FDR < 0.01. Abbreviations: FFB, female flower bud; MFB, male flower bud.

**Figure 6:**
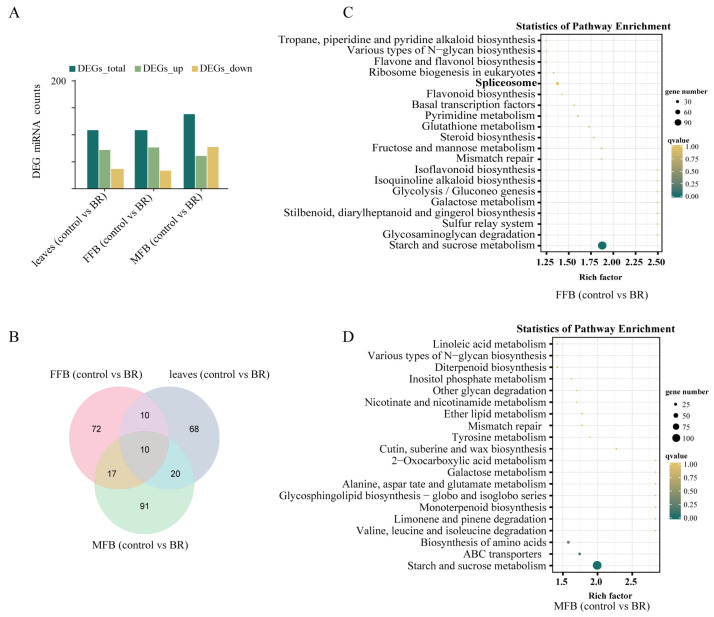
Screening and functional identification of differentially responsive miRNA in female and male walnut flower buds after BR application. (**A**) Number of differentially expressed miRNAs in female and male flower buds after BR treatment (0.05 mg/L). (**B**) Venn diagram showing tissue-specific and overlapping differentially expressed miRNAs among leaves, female flower buds, and male flower buds. (**C**) KEGG pathway enrichment analysis of target genes of differentially expressed miRNAs in female flower buds. (**D**) KEGG pathway enrichment analysis of target genes of differentially expressed miRNAs in male flower buds. Differentially expressed miRNAs were identified with |log_2_(fold change)| ≥ 1 and false discovery rate (FDR) ≤ 0.01. Only pathways with *p* < 0.01 are shown in (**C**,**D**). Abbreviations: FFB, female flower bud; MFB, male flower bud.

**Figure 7:**
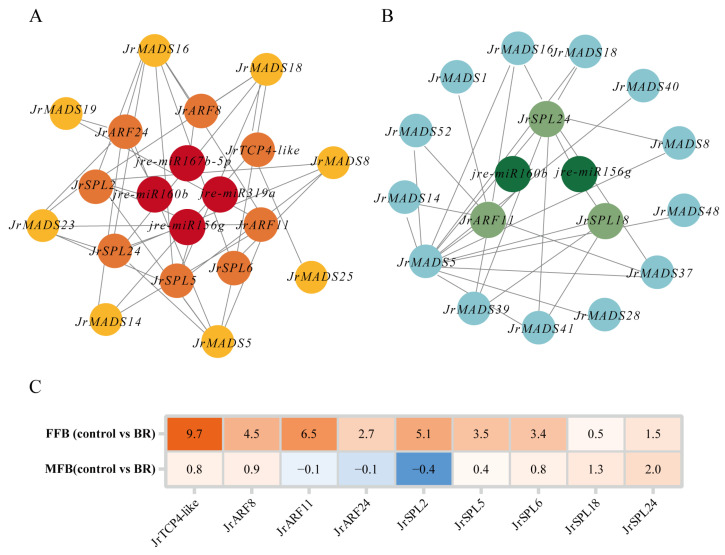
Identification of miRNA-mRNA regulatory modules targeting the MADS-MIKC gene family (**A**) Core miRNA-mRNA modules targeting the MADS-MIKC gene family in female flower buds. (**B**) Core miRNA-mRNA modules targeting the MADS-MIKC gene family in male flower buds. The modules were identified through integrated transcriptomic and miRNA-seq analysis. (**C**) Expression patterns of core differentially expressed genes in female and male flower buds. The color scale represents the log_2_ (fold change) values, with orange indicating up-regulation and blue indicating down-regulation. Differentially expressed genes were identified with |log_2_FC| ≥ 2 and FDR < 0.01. Abbreviations: FFB, female flower bud; MFB, male flower bud.

**Figure 8:**
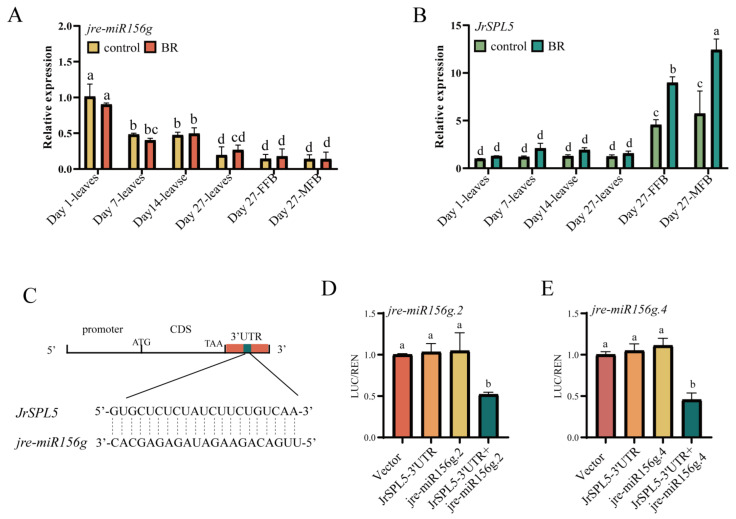
*jre-miR156g* targets and cleaves the 3’-untranslated region of *Jr*SPL5. (**A**,**B**) Relative expression levels of *jre-miR156g* and *JrSPL5* in leaves collected on 17 April (Day 1), 23 April (Day 7) and 30 April (Day 14) of 2025 (Year 3), as well as in leaves, female flower buds and male flower buds collected on 13 May (Day 27). (**C**) Schematic diagram of the *JrSPL5* gene structure. Boxes and lines represent the promoter, coding sequence (CDS) and intron 3’-untranslated region (3’ UTR), respectively. In the schematic diagram, the 3’ UTR is highlighted in orange, and the *jre-miR156g* target site is shown in green. ATG and TAA indicate the start and stop codons. (**D**,**E**) We validate, using a dual-luciferase assay, that *jre-miR156g.2* and *jre-miR156g.4* target and cleave the 3’ UTR region of *JrSPL5*. Data are presented as mean ± standard deviation (*n* = 3). Different letters (a, b, c, d) above the bar graphs indicate significant differences at *p* < 0.05 (one-way ANOVA, Fisher’s LSD test). Abbreviations: FFB, female flower bud; MFB, male flower bud.

## Data Availability

The raw transcriptome and miRNA sequencing data generated in this study are not publicly available at this stage due to their use in ongoing unpublished studies. All processed expression data are provided as Supplementary Materials accompanying this manuscript. The data that support the findings of this study are available from the corresponding author upon reasonable request.

## Supplementary Materials

The following supporting information can be downloaded at: https://www.mdpi.com/article/10.3390/plants15152296/s1. Figure S1. Volcano plots of differentially expressed genes in female and male flower buds. Figure S2. Gene Ontology (GO) enrichment analysis of differentially expressed genes in female and male flower buds. Figure S3. Nucleotide bias of miRNAs in leaves, female flower buds, and male flower buds. Table S1. Weather conditions and sunshine duration during the ten-day periods before and after BR application dates from 2023 to 2025. Table S2. Number of differentially expressed genes in leaves, female flower buds, and male flower buds after BR treatment (0.05 mg/L) (related to Figure 4A). Table S3. Venn diagram showing tissue-specific and overlapping differentially expressed genes among leaves, female flower buds, and male flower buds (related to Figure 4B). Table S4. KEGG pathway enrichment analysis of differentially expressed genes in female and male flower buds (related to Figure 4C,D). Table S5. Fold enrichment of differentially expressed transcription factors (DEG TFs) in major families in female and male flower buds after BR treatment (related to Figure 5A). Table S6. Heatmap showing the expression patterns of differentially expressed MADS-MIKC genes in female and male flower buds (related to Figure 5B). Table S7. Number of differentially expressed miRNAs in female and male flower buds after BR treatment (0.05 mg/L) (related to Figure 6A). Table S8. Venn diagram showing tissue-specific and overlapping differentially expressed miRNAs among leaves, female flower buds, and male flower buds (related to Figure 6B). Table S9. KEGG pathway enrichment analysis of target genes of differentially expressed miRNAs in female and male flower buds (related to Figure 6C,D). Table S10. Core miRNA-mRNA regulatory modules targeting the MADS-MIKC gene family in female and male flower buds (related to Figure 7A,B). Table S11. Expression patterns of core differentially expressed genes in female and male flower buds (related to Figure 7C). Table S12. Primer sequences used for RT-qPCR and dual-luciferase reporter assays to detect JrSPL5 and jre-miR156g expression.

## References

[1] Vahdati K., Arab M.M., Sarikhani S., Sadat-Hosseini M., Leslie C.A., Brown P.J. (2019). Advances in Persian walnut (*Juglans regia* L.) breeding strategies. Advances in Plant Breeding Strategies: Nut and Beverage Crops.

[2] Quan S.W., Niu J.X., Zhou L., Xu H., Ma L., Qin Y. (2019). Stages identifying and transcriptome profiling of the floral transition in *Juglans regia*. Sci. Rep..

[3] Zhang W.E., Li J.J., Zhang W.L., Njie A., Pan X.J. (2022). The changes in C/N, carbohydrate, and amino acid content in leaves during female flower bud differentiation of *Juglans sigillata*. Acta Physiol. Plant..

[4] Gao Y., Liu H., Dong N.G., Pei D. (2012). Temporal and spatial pattern of indole-3-acetic acid occurrence during walnut pistillate flower bud differentiation as revealed by immunohistochemistry. J. Am. Soc. Hortic. Sci..

[5] Hassankhah A., Vahdati K., Rahemi M., Hassani D., Sarikhani Khorami S. (2017). Persian walnut phenology: Effect of chilling and heat requirements on budbreak and flowering date. Int. J. Hortic. Sci. Technol..

[6] Bao C., Chen H., Zhou H., Chen F. (2025). Transcriptomic and metabolomic analysis clarify the molecular mechanisms underlying the formation of sexual and apomictic Persian walnut (*Juglans regia* L.) embryos. Front. Plant Sci..

[7] Li H., Chen L., Liu R., Cao S., Lu Z. (2024). Comparative proteomic analysis of floral buds before and after opening in walnut (*Juglans regia* L.). Int. J. Mol. Sci..

[8] Jin Q., Mo R.L., Chen W.X., Zhang Q.L., Sheng F., Wu C.Y., Zhang R., Luo Z.R. (2022). Identification and comparative analysis of genes and microRNAs Involved in the floral transition of the Xinjiang early-flowering walnut (*Juglans regia* L.). Horticulturae.

[9] Li J.Z., Zhang M.M., Wu J.W., Qin B.T., Liu C.P., Zhang L.J. (2025). Identification and analysis of key miRNA for sex differentiation in hermaphroditic *Juglans mandshurica* Maxim. Plant Mol. Biol. Report..

[10] Li W., Wang Y.M., Qian R.H., Yao Y., Xue X., Chen J., Chu J., Zhu C.H., Xu S.K., Yi C.Q. (2025). Integrative control of plant sex determination: Genes, hormones, and environment. Plant Sci..

[11] Patil R.V., Hadawale K.N., Ramli A.N.M., Wadkar S.S., Bhuyar P. (2023). An Overview of Molecular Basis and Genetic Modification of Floral Organs Genes: Impact of Next-Generation Sequencing. Mol. Biotechnol..

[12] Guerrero-Méndez C., Abraham-Juárez M.J. (2024). Factors specifying sex determination in maize. Plant Reprod..

[13] Chuck G., Meeley R., Irish E., Sakai H., Hake S. (2007). The maize *tasselseed4* microRNA controls sex determination and meristem cell fate by targeting *Tasselseed6/indeterminate spikelet1*. Nat. Genet..

[14] Banks J. (2008). MicroRNA, sex determination and floral meristem determinacy in maize. Genome Biol..

[15] Han L., Li M., Li C., Zhao B., Wang Z., Liu Y., Liu Z., Huang Y., Liu L., Geng H. (2025). *ARF3*-mediated auxin signaling is essential for sex determination in cucumber. Science.

[16] Akagi T., Henry I.M., Tao R., Comai L. (2014). A Y-chromosome–encoded small RNA acts as a sex determinant in persimmons. Science.

[17] Akagi T., Henry I.M., Kawai T., Comai L., Tao R. (2016). Epigenetic regulation of the sex determination gene *MeGI* in polyploid persimmon. Plant Cell.

[18] Adhikari P.B., Kasahara R.D. (2024). An Overview on MADS Box Members in Plants: A Meta-Review. Int. J. Mol. Sci..

[19] Guo C., Fesobi O.P., Zhang Z., Yuan X., Zhao H., Quan S., Niu J. (2025). Identification, Classification of the MIKC-Type MADS-Box Gene Family, and Expression Analysis of Female and Male Flower Buds in Walnut (*Juglans regia*, Juglandaceae). Horticulturae.

[20] Wang T.Q., Khalid E., Mao H.M., Tong Y.H., Xue X.Y., Tang Y.R., Cai L.M., Ming R. (2025). Gibberellin disrupts hormonal homeostasis and anther integrity to trigger sex reversal in spinach. Int. J. Mol. Sci..

[21] Stokes T.S., Croker S.J., Hanke D.E. (2003). Developing inflorescences of male and female *Rumex acetosa* L. show differences in gibberellin content. J. Plant Growth Regul..

[22] Aryal R., Ming R. (2014). Sex determination in flowering plants: Papaya as a model system. Plant Sci..

[23] Akagi T., Henry I.M., Ohtani H., Morimoto T., Beppu K., Kataoka I., Tao R. (2018). A Y-encoded suppressor of feminization arose via lineage-specific duplication of a cytokinin response regulator in kiwifruit. Plant Cell.

[24] Zhu Y.R., Ahmad Z., Lv Y.J., Zhang Y.S., Chen G.D. (2025). Insight into the characterization of two female suppressor gene families: *SOFF* and *SyGI* in plants. Genes.

[25] Boualem A., Troadec C., Camps C., Lemhemdi A., Morin H., Sari M.A., Fraenkel-Zagouri R., Kovalski I., Dogimont C., Perl-Treves R. (2015). A cucurbit androecy gene reveals how unisexual flowers develop and dioecy emerges. Science.

[26] Huang H.-Y., Zhang S., Choucha F.A., Verdenaud M., Tan F.-Q., Pichot C., Parsa H.S., Slavkovic F., Chen Q., Troadec C. (2024). Harbinger transposon insertion in ethylene signaling gene leads to emergence of new sexual forms in cucurbits. Nat. Commun..

[27] Hartwig T., Chuck G.S., Fujioka S., Klempien A., Weizbauer R., Potluri D.P.V., Choe S., Johal G.S., Schulz B. (2011). Brassinosteroid control of sex determination in maize. Proc. Natl. Acad. Sci. USA.

[28] Guo A.H., Nie H.S., Li H.J., Li B., Cheng C., Jiang K.Y., Zhu S.W., Zhao N., Hua J.P. (2025). The *miR3367*-*lncRNA67*-*GhCYP724B* module regulates male sterility by modulating brassinosteroid biosynthesis and interacting with Aorf27 in *Gossypium hirsutum*. J. Integr. Plant Biol..

[29] Yin W., Dong N., Li X., Yang Y., Lu Z., Zhou W., Qian Q., Chu C., Tong H. (2025). Understanding brassinosteroid-centric phytohormone interactions for crop improvement. J. Integr. Plant Biol..

[30] Hassankhah A., Rahemi M., Mozafari M.R., Vahdati K. (2018). Flower development in walnut: Altering the flowering pattern by gibberellic acid application. Not. Bot. Horti Agrobot. Cluj-Napoca.

[31] Aryal D., Alferez F. (2025). Brassinosteroids: Biosynthesis, signaling, and hormonal crosstalk as related to fruit yield and quality. Plants.

[32] Grove M.D., Spencer G.F., Rohwedder W.K., Mandava N., Worley J.F., Warthen J.D., Steffens G.L., Flippen-Anderson J.L., Cook J.C. (1979). Brassinolide, a plant growth-promoting steroid isolated from Brassica napus pollen. Nature.

[33] Cai H., Liu L., Huang Y., Zhu W., Qi J., Xi X., Aslam M., Dresselhaus T., Qin Y. (2022). Brassinosteroid signaling regulates female germline specification in *Arabidopsis*. Curr. Biol..

[34] Lima R.B., Figueiredo D.D. (2024). Sex on Steroids: How Brassinosteroids Shape Reproductive Development in Flowering Plants. Plant Cell Physiol..

[35] Burow K.M., Yang X., Zhou Y., Dilkes B.P., Wisecaver J.H. (2025). A BRASSINOSTEROID INSENSISTIVE 1 receptor kinase ortholog is required for sex determination in *Ceratopteris richardii*. Plant Cell.

[36] Pinto S.C., Mendes M.A., Coimbra S., Tucker M.R. (2019). Revisiting the Female Germline and Its Expanding Toolbox. Trends Plant Sci..

[37] Wang X., Liu J., Shang E., Hawar A., Ito T., Sun B. (2024). Brassinosteroid signaling represses ZINC FINGER PROTEIN11 to regulate ovule development in *Arabidopsis*. Plant Cell.

[38] Clouse S.D., Langford M., McMorris T.C. (1996). A brassinosteroid-insensitive mutant in *Arabidopsis thaliana* exhibits multiple defects in growth and development. Plant Physiol..

[39] Szekeres M., Nemeth K., KonczKalman Z., Mathur J., Kauschmann A., Altmann T., Redei G.P., Nagy F., Schell J., Koncz C. (1996). Brassinosteroids rescue the deficiency of CYP90, a cytochrome P450, controlling cell elongation and de-etiolation in *arabidopsis*. Cell.

[40] Ye Q.Q., Zhu W.J., Li L., Zhang S.S., Yin Y.H., Ma H., Wang X.L. (2010). Brassinosteroids control male fertility by regulating the expression of key genes involved in *Arabidopsis* anther and pollen development. Proc. Natl. Acad. Sci. USA.

[41] Gao Y.J., Zhang Y.L., Wang W.H., Latif A., Wang Y.T., Tang W.Q., Pu C.X., Sun Y. (2025). Protein phosphatase 2A B’α and B’β promote pollen wall construction partially through BRASSINAZOLE-RESISTANT 1-activated cysteine protease gene *CEP1* in *Arabidopsis*. J. Exp. Bot..

[42] Unte U.S., Sorensen A.-M., Pesaresi P., Gandikota M., Leister D., Saedler H., Huijser P. (2003). *SPL8*, an SBP-box gene that affects pollen sac development in *Arabidopsis*. Plant Cell.

[43] Chen X., Zhang Z., Liu D., Zhang K., Li A., Mao L. (2010). SQUAMOSA promoter-binding protein-like transcription factors: Star players for plant growth and development. J. Integr. Plant Biol..

[44] Ma K., Zhao Y., Han L.Q., Gao C.Y., Hu J.F., Guan P.Y. (2024). Genome-Wide analysis of *SPL* gene family and functional identification of *JrSPL02* Gene in the early flowering of walnut. Horticulturae.

[45] Wang C., Zhou M., Yu T., Zhao S., Ruan C. (2026). Non-coding RNA mediated competitive endogenous RNA regulatory network in the sex differentiation of yellowhorn (*Xanthoceras sorbifolium* Bunge.) flowers. Sci. Hortic..

[46] Solar A., Osterc G., Stampar F., Kelc D. (2011). Branching of annual shoots in common walnut (*Juglans regia* L.) as affected by bud production and indol-3-acetic acid (IAA) content. Trees-Struct. Funct..

[47] McGranahan G.H., Voyiatzis D.G., Catlin P.B., Polito V.S. (1994). High pollen loads can cause pistillate flower abscission in walnut. J. Am. Soc. Hortic. Sci..

[48] Kumar S., Sharma N., Sopory S.K., Sanan-Mishra N. (2024). MiRNAs and genes as molecular regulators of rice grain morphology and yield. Plant Physiol. Biochem..

[49] Chakraborty N., Ganguly R., Sarkar A., Dasgupta D., Sarkar J., Acharya K., Burachevskaya M., Minkina T., Keswani C. (2025). Multifunctional role of brassinosteroids in plant growth, development, and defense. J. Plant Growth Regul..

[50] Tepkaew T., Khamsuk O., Chumpookam J., Sonjaroon W., Jutamanee K. (2022). Exogenous brassinosteroids regulate mango fruit set through inflorescence development and pollen fertility. Hortic. Sci. Technol..

[51] Huang W., Tan C., Guo H.W. (2024). Ethylene in fruits: Beyond ripening control. Hortic. Res..

[52] Liu X.F., Zhang Z.H., Sun J.J. (2026). Unraveling the mystery of auxin-promoting femaleness in cucurbits. Hortic. Res..

[53] Xu M., Hu T., Zhao J., Park M.-Y., Earley K.W., Wu G., Yang L., Poethig R.S. (2016). Developmental functions of miR156-regulated *SQUAMOSA PROMOTER BINDING PROTEIN-LIKE* (*SPL*) genes in *Arabidopsis thaliana*. PLoS Genet..

[54] Cui R., Han J., Zhao S., Su K., Wu F., Du X., Xu Q., Chong K., Theißen G., Meng Z. (2010). Functional conservation and diversification of class E floral homeotic genes in rice (*Oryza sativa*). Plant J..

[55] Gregis V., Andrés F., Sessa A., Guerra R.F., Simonini S., Mateos J.L., Torti S., Zambelli F., Prazzoli G.M., Bjerkan K.N. (2013). Identification of pathways directly regulated by SHORT VEGETATIVE PHASE during vegetative and reproductive development in *Arabidopsis*. Genome Biol..

[56] Vyas L., Ojha N., Sharma R., Pathak H., Sudan J. (2023). Functional aspects of miRNA in flower development and flowering. S. Afr. J. Bot..

[57] Liu N., Tu L.L., Wang L.C., Hu H.Y., Xu J., Zhang X.L. (2017). *MicroRNA 157*-targeted *SPL* genes regulate floral organ size and ovule production in cotton. BMC Plant Biol..

[58] Li Y., Deng Y., Qin D., An X. (2025). Study of the *SPL* gene family and *miR156*-*SPL* module in *Populus tomentosa*: Potential roles in juvenile-to-adult phase transition and reproductive phase. Int. J. Biol. Macromol..

[59] Nag A., King S., Jack T. (2009). miR319a targeting of *TCP4* is critical for petal growth and development in *Arabidopsis*. Proc. Natl. Acad. Sci. USA.

[60] Hao K., Wang Y., Zhu Z., Wu Y., Chen R., Zhang L. (2022). miR160: An Indispensable Regulator in Plant. Front. Plant Sci..

[61] Yuan J., Wang X., Qu S.T., Shen T., Li M.J., Zhu L.C. (2023). The roles of *miR156* in abiotic and biotic stresses in plants. Plant Physiol. Biochem..

[62] Ritonga F.N., Xu Y., Cui B., Liu X., Gao J., Li J. (2025). Unraveling the multifaceted roles of *SPL* transcription factors in leaf development. Front. Plant Sci..

[63] Chuck G., Cigan A.M., Saeteurn K., Hake S. (2007). The heterochronic maize mutant *Corngrass1* results from overexpression of a tandem microRNA. Nat. Genet..

[64] Xing S., Salinas M., Höhmann S., Berndtgen R., Huijser P. (2010). *miR156*-targeted and nontargeted *SBP-box* transcription factors act in concert to secure male fertility in *Arabidopsis*. Plant Cell.

[65] Silva G.F.F.E., Silva E.M., da Silva Azevedo M., Guivin M.A.C., Ramiro D.A., Figueiredo C.R., Carrer H., Peres L.E.P., Nogueira F.T.S. (2014). *microRNA156*-targeted *SPL*/*SBP* box transcription factors regulate tomato ovary and fruit development. Plant J..

[66] Chen M., Zhang T.L., Zhang W.B., Wen Y.Z., Ma Z.X., Xi Z.P., Hu C.G., Zhang J.Z. (2025). *miR156*-*SPLs* module regulates flowering and controls plant height by modulating gibberellin biosynthesis in citrus. Plant Biotechnol. J..

